# Dendrimeric Poly(Epsilon-Lysine) Delivery Systems for the Enhanced Permeability of Flurbiprofen across the Blood-Brain Barrier in Alzheimer’s Disease

**DOI:** 10.3390/ijms19103224

**Published:** 2018-10-18

**Authors:** Shafq Al-azzawi, Dhafir Masheta, Anna L. Guildford, Gary Phillips, Matteo Santin

**Affiliations:** 1 Centre for Regenerative Medicine and Devices, School of Pharmacy and Bimolecular Sciences, University of Brighton, Brighton BN2 4GJ, UK; phar.shafaq.kadhim@uobabylon.edu.iq (S.A.-a.); phar.dhafir.qahtan@uobabylon.edu.iq (D.M.); a.guildford@tissueclick.com (A.L.G.); g.phillips@tissueclick.com (G.P.); 2 College of Pharmacy, University of Babylon, Ministry of Higher Education and Scientific Research, Hilla 51002, Iraq; 3 Tissue Click Ltd., BN2 6SJ Brighton, UK

**Keywords:** Alzheimer’s disease, blood-brain barrier, dendrimers, drug delivery system, neurodegenerative disease

## Abstract

Alzheimer’s disease (AD) is a progressive brain disorder and age-related disease characterised by abnormal accumulation of β-amyloid (Aβ). The development of drugs to combat AD is hampered by the lack of therapeutically-active molecules able to cross the blood-brain barrier (BBB). It is agreed that specifically-designed carriers, such as dendrimers, could support the drug penetration across the BBB. The aim of this study was to design biocompatible and biodegradable dendrimeric delivery systems able to carry Flurbiprofen (FP), as drug for AD treatment, across the BBB and liberate it at the target tissue. These dendrons were synthesised using solid-phase peptide synthesis method and characterised by mass spectrometry and fourier-transform infrared spectroscopy (FTIR). The results revealed successful synthesis of dendrons having FP been integrated during the synthesis at their branching ends. Cytotoxicity assays demonstrated the biocompatibility of the delivery systems, whereas HPLC analysis showed high percentages of permeability across an in vitro BBB model for FP-integrated dendrons. Results also revealed the efficiency of drug conjugates on the γ-secretase enzyme in target cells with evidence of eventual drug release by hydrolysis of the carrier. This study demonstrates that the coupling of FP to dendrimeric delivery systems can successfully be achieved during the synthesis of the poly(epsilon-lysine) macromolecules to improve the transport of the active drug across the BBB.

## 1. Introduction

Alzheimer’s disease (AD) is an age-related and neurodegenerative disease (ND) characterised by gradual impairment in memory. It has been reported that the incidence of AD has increased with 47 million people worldwide living with this disease [1]. AD is typified by plaque accumulation of abnormally folded extracellular deposits of Aβ-amyloid (Aβ) and tau proteins in the brain [2]. Aβ species are generated from the amyloid precursor protein (APP) through proteolytic cleavages initiated by β-secretase (BACE-1), with fragments being further cleaved by γ-secretase. These fragments of APP are made up of different Aβ isoforms depending on the number of amino acids [3]. The most prevalent variant isoforms of Aβ are Aβ40 and Aβ42 which, in AD, form dense clumps of deposits surrounding neurons and giving rise to senile plaques of ND [4].

The successful treatment of AD requires drugs that can penetrate the blood-brain barrier (BBB) which is considered the main obstacle in development of new efficacious therapeutics [5]. Indeed, the presence of the BBB prevents the penetration of almost all drugs, genes, and imaging agents to their site of action, making NDs incurable and difficult to be diagnosed [6]. Various approaches have been developed to tackle this issue, including invasive techniques, chemical modifications and enhanced permeability of the endothelial cells of the BBB via endogenous transcytosis [7]. Meanwhile the endogenous transcytosis approach is considered the most favourable one as it enables the drug to undergo internalisation into the brain effectively without remarkable cytotoxicity [8]. Adsorptive mediated transcytosis (AMT) is a well-studied endogenous transport system which allows essential circulating hydrophobic molecules to enter the brain and has been widely used for its potential in drug delivery across the BBB. In AMT, internalisation occurs mostly via a clathrin-mediated, energy-dependent endocytosis following charge interaction between positively-charged molecule and negative charges on the cell membrane [9].

The exploitation of an endogenous transport system that utilises specifically-designed carrier systems is considered one of the most promising and safest strategies for overcoming the BBB [8]. One carrier system that has recently gained attention for delivering such materials is that of dendrimers (or their tree-like structure equivalent, the dendrons) [10]. Dendrimers are hyperbranched polymeric macromolecules that possess unique molecular architecture with well-defined sizes and structures [11]. The high density of their terminal functional groups is the key property in terms of their potential use in drug and gene delivery and contributes to the molecular surface properties which provide multiple attachment sites for integration and loading of drugs or other targeting moieties [12]. Dendrimer-based products have been successfully utilised for cancer therapy and in imaging [13]. It has been found that poly(amido amine) (PAMAM) dendrimer complexes with some nonsteroidal anti-inflammatory drugs (NSAIDs) such as ketoprofen and indomethacin improve the drug permeation through the skin [14]. In oral drug delivery studies, low-generation PAMAM dendrimers have shown to cross cell membranes, presumably through adsorptive endocytosis processes [15]. Furthermore, dendrimer-ibuprofen complexes have been found to enter lung cells more rapidly when compared to drug alone, suggesting that dendrimers can efficiently carry the encapsulated drug into cells [16]. Sadekar et al. (2013) have used PAMAM dendrimer conjugated with the anticancer drug camptothecin to enhance its bioavailability within target tissues [12]. Many studies have utilised dendrimers as drug delivery systems targeting the brain because of their ability to cross the BBB. It was found that a conjugation of the drug doxorubicin to a polyethelenglycol dendrimer enhanced drug transport across the BBB and reduced tumour volume of glioma spheroids in vitro [9,17].

Recently, interest in the effect of NSAIDs, specifically Flurbiprofen (FP), as γ-secretase modulators (GSMs) has grown due to their potential effectiveness in the treatment of AD [18]. FP is able to selectively decrease Aβ42 secretion by subtly altering γ-secretase activity without significant impairment for Notch signalling or other APP processing pathways. FP is supposed to act only on presenilin, the catalytic subunit of γ-secretase which is initially involved in APP cleavage and production of Aβ42 [5]. This action is different from non-selective γ-secretase inhibition by other inhibitor therapies which drastically affects the metabolism of total amyloid proteins which, in turn, regulate various neuronal and synaptic functions [19]. On the other hand, some published data have revealed unsatisfactory results for FP suggesting that targeting γ-secretase is not clinically efficient for AD treatment and that FP improves learning deficits only when administered prior to plaque deposition [20,21]. However, FP is still a recommended medication in reducing the symptoms of AD, particularly at early stages, and it offers neuroprotective effects by preventing mitochondrial calcium overload generated from Aβ toxicity [22,23].

FP is a drug approved by the United States Food and Drug Adminstration and commercially available as both an effective pain reliever and an anti-inflammatory drug and commonly used for rheumatoid arthritis and osteoarthritis [24]. It is a small (244.26 g/mol), poorly water-soluble molecule and a generally well-absorbed drug with 4 h biological half-life and that is fully excreted from the body within 24 h [25]. Therefore, FP was deemed to be a good drug candidate for coupling with a nanocarrier system such as the dendrimers. This choice was motivated by FP low brain permeability bringing the drug concentration at the target tissue site below the required therapeutic concentration (150–250 µM) needed to exert pharmacologically-significant effect on γ-secretase activity [26,27]. Most drugs fail to cross the BBB due to the presence of tight junctions in between the brain endothelial cells which block the paracellular pathway [5]. Indeed, Phase II clinical studies of FP have shown improvement of cognitive functions only in AD patients at the early stage of disease [26,27]. Therefore, the design of a delivery system capable of overcoming the BBB and transport the drug into the brain via alternative pathways such as AMT is advocated. Hence, this study reports a method of synthesis of poly(epsilon-lysine) dendrons that are designed with a molecular root made of a hydrophobic phenylalanine facilitating the penetration of the BBB and the presentation of more than one FP molecule at the ends of the molecular branches.

Low generation (G) 0 and 1 lysine (K) dendrons were synthesised using solid-phase peptide synthesis (SPPS) and successful integrations of FP molecules (G0K-FP and G1K-FP) during the synthesis were achieved as confirmed by mass spectrometry and Fourier-transform infrared spectroscopy (FTIR). The potential cytotoxicity of the studied delivery systems and their BBB penetration potential in a transwell system were tested using immortalised bEnd.3, brain endothelial cells with tight junctions similar to those of the BBB endothelium. Study of cell membrane integrity and of metabolic activity were considered as parameters of potential cell cytotoxicity, while trans-endothelial electrical resistance (TEER) and high performance liquid chromatography (HPLC) analysis were used to assess changes in FP and FP-dendron complex permeability across an in vitro BBB model. In addition, the effect of conjugation on drug activity against its target cells was tested using immortalised cells with an astroglial-like phenotype. The present study showed that the complexed drug still retained the capability of reducing this cellular enzymatic activity. The degradation of complexes and release of drug were also investigated revealing the ability of the carrier to undergo hydrolysis and release the drug. This study demonstrates the successful designing of biocompatible and biodegradable dendrimeric delivery systems with the potential to improve drug transport across the endothelial component of the BBB.

## 2. Results

### 2.1. Characterisation of Flurbiprofen (FP)-Loaded Dendrons

**Figure 1:**
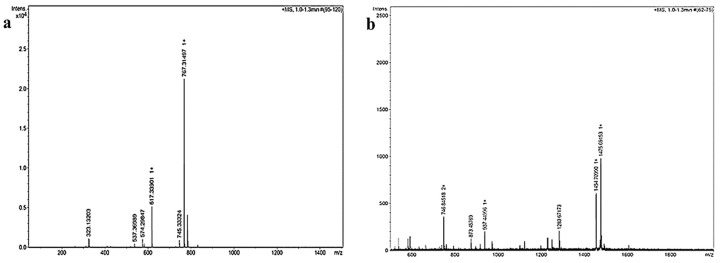
Mass spectra of (**a**) G0K-FP (flurbiprofen) showing a peak at 745.33 represents the exact molecular weight (MW) of product and the highest peak of 767.31 represents the sodium salt. (**b**) G1K-FP showing a peak of 1454.68 representing the exact MW of product and the highest intensity peak of 1475.69 representing the sodium salt in addition to the double charged ion appeared in 746.84 *m*/*z*.

The mass spectra demonstrated the successful loading of FP to both G0- and G1-dendron with peaks corresponding to the expected molecular weight (MW) (745.33 Da for G0K-FP and 1454.7 Da for G1K-FP) as presented in Figure 1a,b, respectively. Other peaks appearing in the spectra result from the ionisation of the molecule and the solvent or machine noise, as well as, the sodium salts formed due to interaction of ions with glass vessels [28].

**Figure 2:**
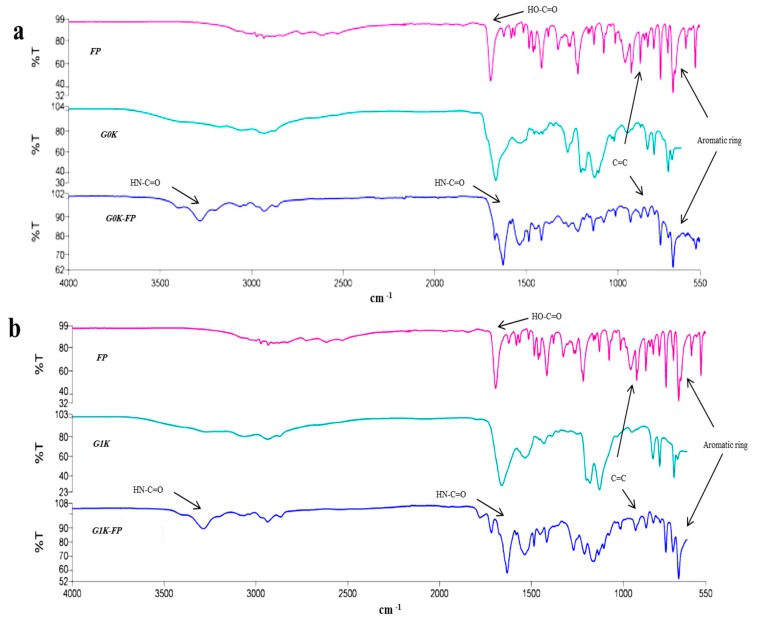
Fourier-transform infrared spectroscopy (FTIR) spectra of (**a**) FP alone, G0K and G0K-FP, (**b**) FP alone, G1K and G1K-FP. The spectra show shifting of peaks due to formation of peptide linkages at 3200 and 1646 cm^−1^. FP spectrum shows a strong carbonyl band seen at 1700 cm^−1^, related to its COOH functional group that disappeared in the G0K-FP and G0K-FP spectra due to amide linkage formation. Appearance of peaks at 782 cm^−1^ corresponded to the aromatic ring of FP with its C=C peak at 910 cm^−1^ in the G0K-FP and G1K-FP spectra confirms the FP attachment to the dendrons.

FTIR analysis revealed a shift in peaks due to the formation of an amide linkage at 3200 cm^−1^ and 1640 cm^−1^ that also confirm the attachment of drug to both G0- and G1-dendrons (Figure 2).

### 2.2. Cytotoxicity and Biocompatibility Studies

**Figure 3:**
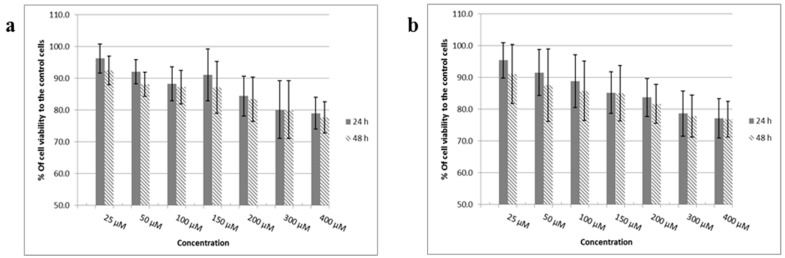
MTT (1-(4,5-Dimethylthiazol-2-yl)-3,5-diphenylformazan thiazolyl blue tetrazolium) results after 24 and 48 h treatment of bEnd.3 cells with: (**a**) G0K-FP and (**b**) G1K-FP. The absorbance was measured and the cell viability was calculated as a percentage of absorbance in relation to control cells. The data represent mean ± SD (standard deviation) of *n* = 6.

MTT (1-(4,5-Dimethylthiazol-2-yl)-3,5-diphenylformazan thiazolyl blue tetrazolium) assays were carried out after 24 and 48 h treatment of post confluent bEnd.3 cells with increasing concentrations of FP-loaded dendrons. The results demonstrated cell viability in excess of 70% in relation to the control untreated cells (Figure 3) and values still within acceptable cytotoxic range (International-Standards 2009) suggesting that drug-loaded dendrons were not toxic to these cells even at the highest concentration used (400 µM). In addition, there was no significant differences (*p* > 0.05) between the corresponding concentrations after 24 or 48 h exposure.

**Figure 4:**
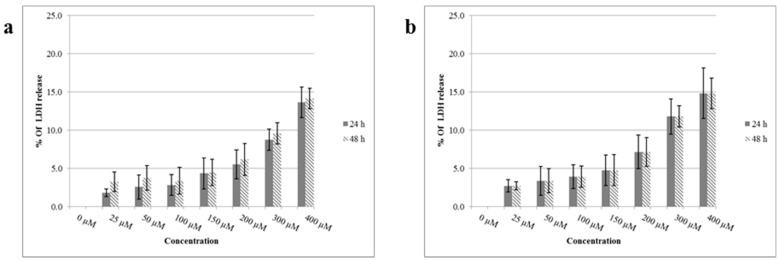
Lactate dehydrogenase (LDH) results after 24 and 48 h treatment of bEnd.3 cells with: (**a**) G0K-FP and (**b**) G1K-FP. The absorbance was measured and the LDH release of each was calculated as a percentage of absorbance in relation to untreated, complete lysis control cells with significantly different to the positive control (*p* < 0.001). The data represent mean ± SD of *n* = 6.

The results of the lactate dehydrogenase (LDH) assay supported the findings of the MTT assay with low values of LDH release after 24 and 48 h treatment of bEnd.3 cells with FP-loaded dendrons in a range of concentration up to 400 µM. Both results are significantly different (*p* < 0.001) to the positive control (100% complete lysis) with no significant differences (*p* > 0.05) in between each other (Figure 4).

### 2.3. Penetration of Drug-Integrated Dendrons Across an in Vitro Model of The Blood-Brain Barrier (BBB)

**Figure 5:**
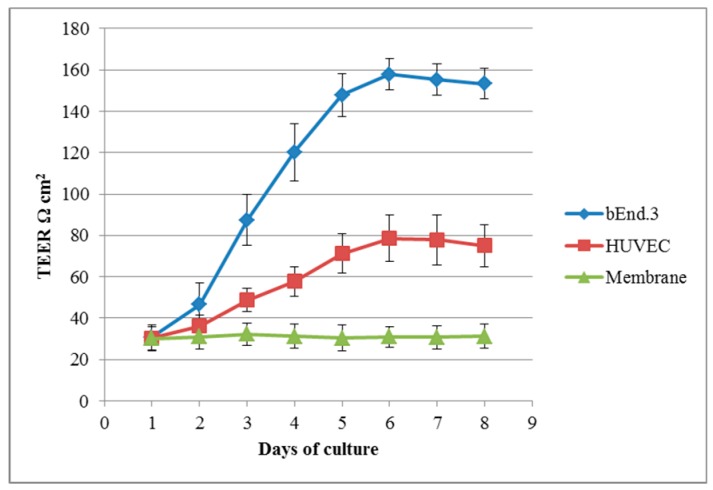
Trans-endothelial electrical resistance (TEER) measurement of the cultured bEnd.3 cells, human umbilical vein endothelial cells (HUVEC) and cell-free inserts (membrane).

Penetration of free drug and drug-loaded molecules across the bEnd.3 monolayer culture using a Transwell system was carried out at the day 6 of culturing. Trans-endothelial electrical resistance (TEER) values of cultured bEnd.3 increased to a highest reproducible value at day 6 (at confluence) in comparison to the cultured human umbilical vein endothelial cells (HUVEC) and the cell-free inserts values (Figure 5).

**Figure 6:**
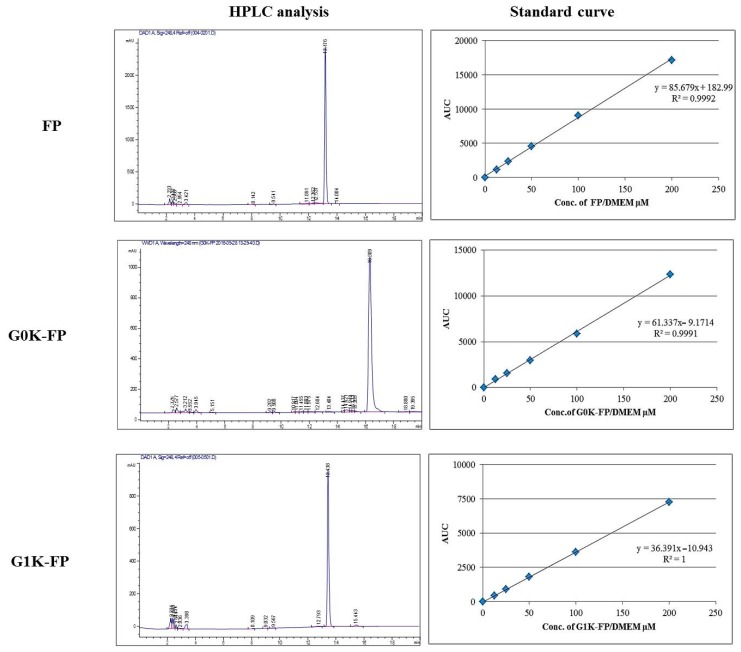
HPLC analysis and corresponding standard curve of FP, G0K-FP, and G1K-FP.

The penetration analysis was performed using HPLC after 1 and 4 h of application of the materials to the apical surface of the cells. A series of dilutions of FP, G0K-FP and G1K-FP in (phenol-free) Dulbecco’s modified eagle’s medium (DMEM) solution were analysed and the relative standard curves constructed (Figure 6). For free drug, the amount that permeated, as measured by drug appearing in the basolateral chamber, was not more than 8.5% of applied concentration after 4 h. The percentage of permeability of G0K-FP after 1 and 4 h were 5.3 and 12.5%, respectively, of that uploaded in the apical chamber, with no significant differences (*p* > 0.05) found from that of the free drug of corresponding time of incubation. The calculated amounts of the G1K-FP were 4.3 and 14.8% of the initial applied amount, which are significantly different (*p* < 0.05) from that of the free FP in both time intervals (Table 1). It is worth mentioning that the G0K-FP bearing 2 more moles of drug moieties while G1K-FP having 4, in turn this could increase the bioavailability of drug upon dissociation at target tissue.

### 2.4. Evaluation of Drug-Integrated Dendrons Activity on γ-Secretase Enzyme

As illustrated in Table 2; the calculated amount of γ-secretase from set of C6 glial control cells were in an average of 28.4 pg/mL, while treated cells with FP significantly decreased (*p* < 0.05) the levels to 8.2 pg/mL. The results showed that the cells treated with G0K-FP significantly decreased (*p* < 0.05) the amount of the enzyme to an average of 12.7 pg/mL when compared to that of the control, and not significantly different (*p* > 0.05) from FP. The analysis also found a significant decrease (*p* < 0.05) in γ-secretase activity levels for the set of cells that were incubated with G1K-FP in comparison to both control and free drug with an average value of 16.0 pg/mL.

### 2.5. Degradation Investigation

**Figure 7:**
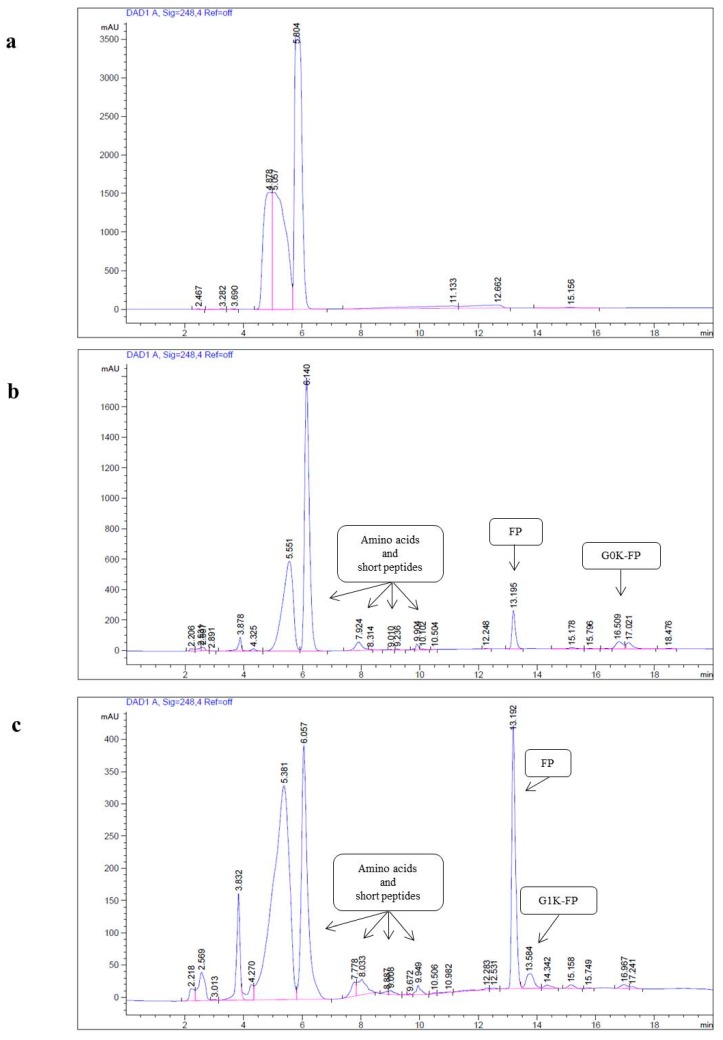
HPLC analysis of: (**a**) Acidic buffer only (background); (**b**) G0K-FP and (**c**) G1K-FP in an acidic buffer (phthalate) solution of pH 4.5. The peaks at the beginning of the HPLC run, before 6 min, are related to the buffer (background), whereas at 13.1 min elution represents free drug. The remaining detected peaks are likely representing the amino acid residues used during synthesis of the dendrons or the shorter peptides that formed due to the breakdown.

To test the hydrolysis of the synthesised macromolecule and the dissociation of the attached drug in an environment mimicking the inflamed tissue typical of AD, acidic buffer (pH 4.5) was used. The results of the qualitative HPLC analyses of G0K-FP and G1K-FP demonstrated that different peaks appeared in each analysis signifying the dissociation of molecules into various products. The peaks at 13.1 min elution are clearly representing the drug release, while those before 6 min of run are related to the buffer (background). Alternatively, the remaining detected peaks that are not related to the drug-bound carriers are most likely representing the amino acid residues that have been used in the assembly of the dendrons including lysine, phenylalanine, or to the shorter peptides that formed due to the hydrolysis. These hydrolysed products have been detected with a reduction in retention time in the HPLC column which is due to their lower MW and hydrophobicity (Figure 7).

## 3. Discussion

The effective therapy in AD and other NDs remain a huge unsolved problem [29] as many therapeutics are unable to permeate through the BBB. Several strategies to improve the delivery of molecules to the brain have been developed including local injection or BBB opening and enhancing the permeability and targeting delivery [30]. Accordingly, the endogenous transport systems available on the BBB is the most potential and safest way to deliver molecules into the brain. Indeed, certain peptides and some macromolecules can pass through BBB via transcytosis mechanisms either by AMT or receptor mediated trancytosis [31]. Generally, endocytosis in AMT is promoted by the interaction of the cationic molecule with phospholipids and the glycocalyx at membrane. In this process, the formed vesicle (from molecule-membrane interaction) circulates across the cytoplasm, and eventually releases the content into the abluminal side by exocytosis [9]. Currently, dendrons offer an important non-invasive strategy in drug delivering and targeting, arising from their structural characteristics. Dendrons provide the possibility of multi-functionalisation, as well the ability to act as intra-cellular drug carriers and cross biological barriers via adsorption [32]. In addition, their biocompatible and biodegradable properties make them successful drug shuttles [33]. In the present study, dendrimeric complexes with low generations of molecular branching were used as carrier systems able to avoid the formation of complexes with a hydrodynamic radius too large to penetrate the cells while ensuring sufficient drug loading capacity. In addition, the design of the complex with a molecular root presenting a relatively hydrophobic amino acid, the phenylalanine, was deemed appropriate to facilitate the penetration of the phospholipid bi-layer of the BBB endothelial cell membrane.

FP is one of GSMs that have emerged to the forefront of AD therapy as a profound disease modifying agent, especially at early stages [4]. Despite their unclear mechanism of action, GSMs are selectively reducing the production of pathogenic Aβ42 isomer, yet without affecting the total amount of Aβ formed [18]. On the other hand, FP failed in clinical trials to improve poor cognition and other Alzheimer’s symptoms due to the existence of BBB [4,26]. It has been found that the drug’s low permeability across the BBB reduces its levels at the brain target cells below its pharmacological doses [24,26]. Hence, in this work branched lysine-dendrons (G0 and G1) carriers were designed to load the drug and enhance its BBB permeability. To achieve this purpose, microwave-based SPPS was employed which ensured a high percentage of productivity and purity of the peptidic carrier [34].

The data resulted from mass spectra analysis for FP-integrated dendrons indicated the successful synthesis and drug loading via amide linkage for both by giving the expected molecular weight (Figure 1). The formation of the amide linkage is dependent upon the side-chain protectors of amino acids which can ensure the chemical reaction of Fmoc in the site of interest only. Therefore, using the appropriate Fmoc type prevents unwanted reactions that could result in the formation or incorporation of dipeptide derivatives. Subsequently, it can help in the purification of the final product due to smaller amounts of secondary products [35]. The peptide linkage formation was confirmed by the FTIR results (Figure 2), this bond is important for the later biodegradability of the peptide and release of drug.

The success of any carrier systems or biomaterials to be used in drug or gene delivery is the appropriate biocompatibility and biodegradability properties [33]. Concentrations of FP-integrated dendrons up to 400 µM did not affect cell viability after 24 h and 48 h below 77%, signifying no considerable effect on mitochondrial function when compared to control cells (Figure 3). Furthermore, they did not show any significant effect on the cell membrane according to the cell lysis results of LDH assays as results were far lower than that for positive control where cell membrane integrity was deliberately disrupted (Figure 4). These findings indicate the biocompatibility of these synthetic products and are consistent with other studies that showed no cytotoxic impact when investigating the low generation of dendrimers. Previous studies have stated that the dendron cytotoxicity appears with higher concentration and generation which is attributed mainly to the end group present on its periphery [36,37]. Generally, amine-terminated dendrimers display concentration-dependent toxicity [32,38]. These positively-charged groups interact with the negatively-charged cell membranes causing cell membrane damage [32] thus increasing LDH release and affecting the mitochondrial function and the overall cell viability. For this reason, cell activity and membrane integrity are affected only upon exposure to relatively high concentrations of both FP-dendrons, but still within acceptable ranges indicating that these systems can be used safely as carriers for drug delivery.

To assess the permeability of molecules towards the BBB, a valid model mimicking primarily the endothelial tight junctions should be used. The brain endothelial cell line used in the study (bEnd.3) was cultured on microporous Transwell^®^ inserts as it has previously been shown to have great potential as a BBB model and widely utilised for drug transport studies [39,40]. TEER measurements in bEnd.3 were found greater than that of HUVEC and membrane only suggesting a sufficient formation of tight junctions to produce an integral cell layer. This finding is in agreement with other studies that have proved the validity of this cell line for simulation of the in vivo situation [41,42,43].

The permeability examination of free drug and drug-complexed dendrons across the in vitro BBB model showed improvement when drug attached to the carrier systems (Table 1). Free FP was found to penetrate less than 9% of its initial payload concentration; a finding to some extent similar to previous study results which revealed that the FP diffusion through bEnd.3 cells monolayer was approximately 10%, a level that is not sufficient to induce its pharmacological effect [5]. Alternatively, an increase in penetration was observed for FP-conjugated dendrons (G0 and G1) to be 12–14% when compared to free drug. This can be attributed to both the positive charges of the dendron amine groups and the hydrophobic character of the phenylalanine root which are likely to facilitate the complex cellular transport. Indeed, earlier investigations found that the cationic dendrimers such as poly-lysine, poly-propylene imine, and PAMAM could interact electrostatically with negative charges of the biological membranes leading to profound applicability for intracellular drug delivery [32,44]. As documented previously, folic acid has been efficiently delivered to the target when conjugated with polyethyleneglycol 5000-dendrimer [45]. Furthermore, paclitaxel (anticancer drug) when conjugated with PAMAM dendrimer demonstrated 12-fold greater permeability across brain endothelial cells and Caco-2 cell monolayers than paclitaxel alone [46]. In addition, the permeation of ketoprofen and diflunisel through skin tissue improved when integrated into PAMAM dendrimers [14], while G0 PAMAM dendrimer conjugated to naproxen showed high permeability across intestinal Caco-2 cells [47,48]. Cationic peptide can enter cells and mostly undergo endocytosis through different mechanisms and it is assumed that they possibly undergo AMT to cross the BBB [31]. However, the transcytosis in cationic carriers may need longer time for exocytosis than other pathways thus making them suitable for a sustained drug delivery. This proposition is supported by many scientists suggesting that dendrimers enhanced uptake with consequent release of cargo into target cells [32,49]. It has been observed that ibuprofen-dendrimers complexes rapidly undergo endocytosis by cells and subsequently sustain the release of the drug [16]. Likewise, studies have demonstrated that the conjugate of PAMAM with erythromycin or azithromycin have also high drug payload and sustained release [50].

The moderate penetration of the complexes observed in this study could be ascribed to the low generation of dendrons used. Previous studies have observed that cationic dendrimers with different branching generations (G0-G4) differ in their permeability across Caco-2 cell monolayers and increasing with the branching generation [51]. In another study, G3-NH_2_ dendrimer was found to have a lower rate of cellular entry when compared with G4-NH_2_ due to fewer surface charges [52]. At the same time, it is also worth reiterating that the toxicity of dendrimers increases with the generation and size.

Even if the percentage of permeability might only moderately increase with higher branching generations, the quantity of drug conjugated within dendrons (2 more in G0 and 4 more in G1) is double for each branching generation. Hence, the carrier design should take into account penetration efficiency, cytotoxicity and drug payload.

Despite permeability was significantly improved by the complexation of FP with the dendrimers, the conjugation of drug to the dendronised carrier might negatively influence its pharmaceutical activity. For this reason, C6 glial cells were cultured to investigate the activity of the FP-loaded dendronised carrier systems against γ-secretase enzyme activity.

The C6 cell line possesses most of the regulatory control mechanisms and phenotypic features of the neuroglial cells and in particular those of astrocytes. It has been used in many neurological studies as a model that mimics the in vivo phenotype or primary brain cell culture for different research aspects [53,54] and in particular for its ability to express the gamma secretase enzyme that is involved in the amyloid protein precursor cleavage leading to amyloid formation [5]. The results generated from the quantification of the γ-secretase enzyme activity showed that the drug-conjugates still retained the drug action in reducing the activity of γ-secretase of C6 glial cells. However, they were not as active as the free drug, possibly due to the steric hindrance. Several studies have previously provided a line of support to this explanation in which some therapeutic agents lacked their efficiency upon conjugation with a carrier due to steric hindrance [55,56]. A variety of chemistries have been employed to design the delivery system capable of attaching drugs covalently via spacer or biodegradable linkage, such as lysine, to ensure free drug release and preserve its activity [57].

The amide linking between the drug and dendron is the key for the degradability and release of drug as it is hydrolysed enzymatically in the cell. The complexation via hydrolytically labile linkages, such as amides, is more suitable for a better control over drug release and targeting than that can be achieved by encapsulation/electrostatic complexation [17,58]. The structure of the carrier system and the drug is the most important factor that determines the type of reaction and facilitates the chemical conjugation via formation of the functionalities such as amide, ester, acetyl-hydrazone, or disulfide groups [57]. It is noteworthy to mention that FP molecular structure containing carboxyl group facilitated its coupling to dendron via amide linkage. The results of the present investigation assessing the degradation of the drug/carrier complex in acidic conditions, similar to those in vivo, highlighted that only negligible amounts of original integrated molecules remained not degraded. Most of the drug molecules were liberated in the buffer solution alongside with numerous amino acids and small peptides residues. These outcomes suggest that the in vivo administration of the dendronised FP can, in the long-term, lead to the releasing of the free fully active drug. The findings of this study are consistent with a previous study of doxorubicin-aminoethyl polyacrylamide conjugate resulted in releasing doxorubicin via amide linkage cleavage at pH 4.0 (acidic pH encountered in lysosomal enzyme) in less than 24 h and in more than 96 h at pH 7.5 (physiological pH) [59]. Further study has found that cationic G2 and G3 dendrons biodegraded into their building monomers using acidic buffer solution with a rate decreasing with the higher generation number [60]. Our results are also supported by previous finding of methotrexate coupling to poly-lysine polymer via amide linkage, which revealed full drug pharmacological action signifying degradation of linkage as the action needs drug detachment from polymer [55]. Finding from several studies have also demonstrated that linkages in polyamide dendrimers, in comparison to other types of linkages, have higher stability due to relatively lower rate of hydrolysis in blood circulation [61]. Direct amide linking of naproxen with PAMAM dendrimer showed higher chemical stability in plasma and liver homogenate and in different pH buffers including 7.4 and 1.2 than that of ester linking [52]. This feature is necessary to minimise the non-specific interaction of dendrimers with systemic circulation and ensuring that the drug-loaded dendronised system will not be cleared off very rapidly, but rather achieve the delivery of therapeutic doses to the target cells.

The failure of phase II clinical trials for AD patients treated with FP-based drugs (i.e., Flurizan) or other γ−secretase inhibitors (e.g., Semagagestat) has been ascribed to the poor drug penetration across the BBB that demands the administration of high and toxic doses not only failing to treat AD, but also leading to adverse side effects [62,63]. It has been advocated that an improved BBB penetration could allow a treatment at lower and repeated doses [63].

The present work demonstrates that dendrimers integrating FP in their structure can be synthesised with high degree of purity. The hyperbranched carrier increases the drug payload, improves the penetration across endothelia with particularly impermeable tight junctions and allows a gradual release of the γ-secretase inhibitor to the target cells. Although the carrier-coupled drug shows a reduced inhibitory effect towards the γ-secretase enzymatic activity, its gradual release from the carrier has the potential to produce a protracted therapeutic effect. Therefore, the combination of an increased BBB penetration and a longer therapeutic activity has the potential to reduce adverse reactions and improve the efficacy of FP-based treatments of AD.

## 4. Materials and Methods

### 4.1. Synthesis of Delivery Systems

**Figure 8:**
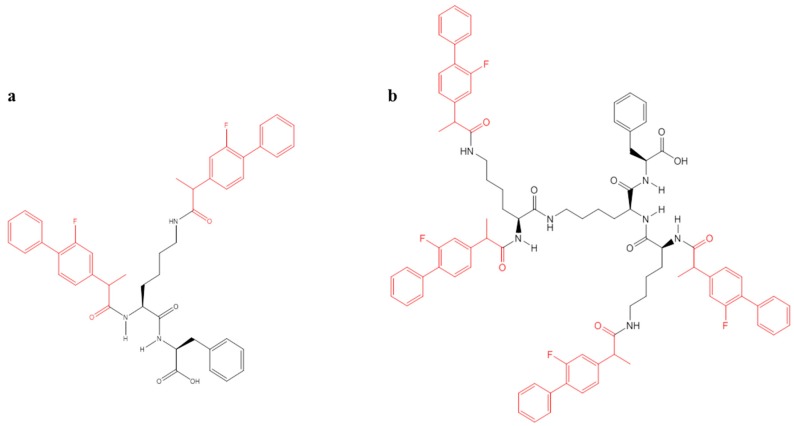
Chemical structure of (**a**) G0K-FP with MW 745 Da, (**b**) G1K-FP MW: 1454.68 Da (using ChemDraw Professional 15).

Generation 0 and 1 lysine dendrons were synthesized with a phenylalanine root using SPPS by the aid of microwave synthesizer (Biotage Initiator, Hengoed, UK). The FP molecules were attached during synthesis at the amino ends by amide linkages to produce G0K-FP (chemical formula: C_45_H_45_F_2_N_3_O_5_ with MW: 745.3 Da), and G1K-FP (chemical formula: C_87_H_91_F_4_N_7_O_9_, with MW: 1454.6 Da) (Figure 8). The assembly started on a Tentagel resin (0.5 g) using 0.4 mmol of phenylalanine (Fmoc-Phe-OH) as a core and lysine (Fmoc-lys(Fmoc)-OH) for G0, and 2 more Fmoc-lys(Fmoc)-OH (Novabiochem, UK) in case of G1.

The synthesis process including coupling, deprotection and cleavage was performed according to a previously discussed method [64]. FP molecules were coupled before cleavage as one molar for each molar NH_2_ end branch, 2 moieties in G0 and 4 in G1. After cleavage, the mixture was filtered through a glass wool and collected in chilled diethyl ether to be centrifuged and washed 3 times, then the precipitant freeze dried [65]. In order to remove any impurities and undesired by-products, Zeba spin desalting columns (Fisher scientific, Southampton, UK) were used and the final pure product was used for subsequent experiments.

### 4.2. Characterisation of The FP-Loaded Dendrimeric Delivery Systems

#### 4.2.1. Mass Spectrometry (MS)

The dried powder of FP-loaded dendrimeric delivery systems were characterised by electrospray/ionisation-time of flight (ESI-TOF MS) (Bruker Daltonics, Coventry, UK) at high voltage (4 kV). In electrospray/ionisation mode, samples mass sample mass (*m*/*z*) gives rise to multiple charged related-ions typically labelled with a number of charges (n) as (MW + nH) n+, in which H = mass of proton (1.008 Da).

#### 4.2.2. Fourier Transform Infra-Red (FTIR)

FTIR spectroscopy (Perkin Elmer Spectrum 65, Llantrisant, UK) was used to investigate the structural and functional group changes in each of the synthesised molecules. A few milligrams of the dried sample was used with a 32-scan run in a 550–4000 cm^−1^ range.

### 4.3. Preparation of Cell Lines

The immortalised brain endothelial cell line, bEnd.3, Dulbecco’s modified eagle’s medium (DMEM), human umbilical vein endothelial cells (HUVECs) and C6 glial cells were obtained from ATCC (USA). The bEnd.3 cells were cultured, according to ATCC-product sheet instruction, in DMEM containing 10% foetal bovine serum (FBS) and 1% of 500 U/mL Penicillin/Streptomycin (Gibco, Gaithersburg, MD, USA). Cells were seeded at a density of 5 × 10^4^ cells per cm^2^ in 24-well plates then incubated at 37 °C and 5% CO_2_. The HUVECs were seeded according to the supplier’s recommendations in endothelial basal medium (F-12K) consisting of 0.05 mg/mL endothelial cell growth supplement, 0.1 mg/mL heparin (ATCC, Manassas, VA, USA) and 10% FBS.

C6 glial cells were cultured in a complete growth medium made according to the ATCC-product sheet by addition of FBS to a final concentration of 2.5% *v*/*v* and horse serum (Gibco, Germany) to a final concentration of 15% *v*/*v* to F-12K medium. The C6 glial cells seeded in a density of 5 × 10^4^ cells per cm^2^ and incubated at 37 °C and 5% CO_2_.

### 4.4. Cytotoxicity and Biocompatibility Assays

Experiments were performed when cells reached confluency and a range of concentrations (25 to 400 µM) of each G0K-FP and G1K-FP were used in each experiment. MTT (Sigma Aldrich, Gillingham, UK) assay was used to measure cell viability [66] after 24 and 48 h treatment exposure. The absorbance values were measured at a wavelength of 540 nm in a spectrophotometer (Thermo Multiskan Ascent, Rochford, UK) and were expressed as percentage of the untreated control cells.

LDH assay provides an indication of loss of cell membrane integrity and was measured using a Promega CytoTox96^®^ non-radioactive cytotoxicity assay kit (UK) after 24 and 48 h treatment. Absorption was read spectrophotometrically at 492 nm and converted to a percentage of the total LDH released from the positive control (untreated cells with complete lysis).

### 4.5. Examination of FP-Loaded Dendrimeric Delivery Systems Penetration Across the in Vitro BBB Model

The lyophilised powders of synthesised molecules (G0K-FP and G1K-FP) as well as free drug (FP) were dissolved in the FBS-free; phenol-free culture media in a concentration of 200 µM. Quantitative analysis of drug and drug-attached dendrons penetration was conducted using HPLC (Agilent technology/1260 infinity, Stockport, UK). The analysis was carried out using a hydrophobic C18 column (150 × 4.6 mm) and a UV-detection wavelength 248 nm which is the λ_max_ of FP [5]. The gradient of eluent of the mobile phase was run from 75:25 to 25:75 of water: acetonitrile over 20 min. The standard curve for each molecule was obtained to calculate the percentage of transported amount across the in vitro BBB model.

To establish an in vitro BBB model, bEnd.3 cells were cultured on microporous membrane (0.4 µm pore size) of 12-Transwell inserts (Fisher Scientific) with complete culture media. TEER values between apical and basolaterl chambers was measured using an Evom voltometer (Sarasota, FL, USA) to determine the day of maximum values indicating tight junction formation. The measurements were taken up to day 8 of culturing and compared to the results of HUVECs which were also cultured into Transwell-clear inserts. Cell-free Transwell inserts were included to exclude the resistance related to the inserts membrane alone. The apical chambers of bEnd.3 culture were then treated with 200 µM of each samples. After 1 and 4 h incubation, 200 µL samples were taken from the basolateral chambers and percentage of permeability calculated according to the following [39]:

*P_Material_* % = ((*C_Acceptor_* × *V_Acceptor_*)/(*C_Donor_* × *V_Donor_*)) × 100% (1)

where *P_Material_* %: the percentage of permeability of the tested material, *C_Acceptor_*: the concentration of the tested material in basal chamber, *V_Acceptor_*: the volume of the culture media in the basal chamber, *C_Donor_*: the initial concentration of the tested material in the apical chamber, *V_Donor_*: the volume of the culture media in the apical chamber.

### 4.6. Evaluation of Drug-Integrated Dendrons Activity on γ-Secretase Enzyme

The investigation is based on sandwich enzyme-linked immune-sorbent assay (ELISA) technology using γ-secretase enzyme kit (Abbexa Ltd., Cambridge, UK). C6 glial cells were cultured in 24-well plates at density 5 × 10^4^ cells per cm^2^, and at confluence, they were treated with 200 µM FP, G0K-FP, or G1K-FP dissolved in F-12K media. Wells containing untreated C6 cells were also included as a control. After 4 h incubation, cells were washed with PBS and lysed via freezing to −20 °C and thawing to room temperature 3 times. The well plates were centrifuged at 1500× *g* for 10 min to remove any cellular debris and the supernatant was collected for assaying the γ-secretase activity (according to kit instructions). The collected samples were used immediately to avoid any degradation and denaturalisation. The standard curve of γ-secretase standards (supplied with kit) was constructed and the concentration of γ-secretase was calculated for each sample.

### 4.7. Degradation Investigation

The dendron-FP complexes were analysed for the hydrolysis of the amide linkage that coupled amino acids residues, as well the drug. The hydrolysis of the synthesised complex was evaluated in a physiological condition using an acidic solution to mimic the lysosomal pH conditions that are likely to be found in an inflamed tissue such as that of AD-affected brain tissue.

The acidic buffer solution was prepared according to supplier’s instructions, by dissolving an acidic buffer (phthalate) tablet (Thomas Scientific, Swedesboro, NJ, USA) in 100 mL of distilled water and the pH was adjusted to 4.5. G0K-FP and G1K-FP were incubated at 37 °C in the acidic buffer solution at a concentration of 200 µM. Samples were then collected after 24 h incubation and filtered with a 0.22 µm filter before analysis by HPLC. HPLC analysis was performed for free FP, G0K-FP and G1K-FP using the same gradient of eluent of the mobile phase mentioned above. Buffer alone samples also included as a background.

### 4.8. Statistical Analysis

Mean values were calculated for the number of readings (n) in each experiment and the error bars refer to the standard deviation (SD). Results were statistically analysed using one-way ANOVA with Tukey’s tests. Significant difference was identified by a *p* value < 0.05.

## Figures and Tables

**Table 1:** Permeability % of flurbiprofen (FP) and FP-loaded dendrons across an in vitro blood-brain barrier (BBB) model.

Molecule	Time	No. of Readings	% Of Permeability (Mean ± SD)	*p* Value to FP	*p* Value to G0K-FP
FP	1 h	6	2.71 ± 1.52		
	4 h	6	8.50 ± 1.32		
G0K-FP	1 h	6	5.31 ± 2.73	>0.05	
	4 h	6	12.48 ± 3.42	>0.05	
G1K-FP	1 h	6	4.32 ± 1.05	<0.05	>0.05
	4 h	6	14.79 ± 2.06	<0.05	>0.05

**Table 2:** Quantification of γ-secretase enzyme after exposure to free FP, G0K-FP and G1K-FP.

Molecule	No. of Readings	γ-Secretase Enzyme Concentration pg/mL (Mean ± SD)	*p* Value to FP	*p* Value to Control
Control	6	28.43 ± 8.32	<0.05	
FP	6	8.20 ± 3.80		
G0K-FP	6	12.69 ± 4.85	>0.05	<0.05
G1K-FP	6	16.04 ± 3.18	<0.05	<0.05

## Abbreviations

AD	Alzheimer’s disease
AMT	Adsorptive-mediated transcytosis
Apo-E	Apolipoprotein E
APP	Amyloid precursor protein
Aβ	β-amyloid
BBB	Blood brain barrier
bEnd.3	Immortalised brain endothelial cells
CNS	Central nervous system
DMEM	Dulbecco’s modified eagle’s medium
ELISA	Enzyme-linked immune-sorbent assay
FBS	Foetal bovine serum
FP	Flurbiprofen
FTIR	Fourier transform infra-red
G	Generation
G0K-FP	Generation 0 lysine dendron-2Flurbiprofen
G1K-FP	Generation 1 lysine dendron-4Flurbiprofen
GSMs	γ-secretase modulators
HPLC	High performance liquid chromatography
HUVEC	Human umbilical vein endothelial cells
LDH	Lactate dehydrogenase
*m*/*z*	Mass to charge ratio
MTT	3-(4,5-Dimethylthiazol-2-yl)-3,5-diphenylformazan
MW	Molecular weight
ND	Neurodegenerative
NSAIDs	Nonsteroidal anti-inflammatory drugs
PAMAM	Poly amido amine dendrimers
SD	Standard deviation
SPPS	Solid phase peptide synthesis
TEER	Transepithelial electrical resistance
